# Executive dysfunction and its impact on quality of life in adults with ADHD

**DOI:** 10.1192/j.eurpsy.2026.11135

**Published:** 2026-07-31

**Authors:** B. A. Oroian, A. Szalontay

**Affiliations:** 1 “Grigore T. Popa” University of Medicine and Pharmacy; 2 “Socola” Institute of Psychiatry, Iasi, Romania

## Abstract

**Introduction:**

Executive dysfunction is a major source of difficulty for adults with ADHD and often becomes more troublesome with age as life demands increase. Beyond inattention and hyperactivity, many struggle with planning, organization, and emotional control, challenges that affect work, relationships, and overall well-being. Although these cognitive and emotional aspects are well recognized, their combined impact on quality of life remains less explored.

**Objectives:**

To examine the relationship between self-reported executive dysfunction and quality of life in adults with ADHD, and to identify which executive domains most strongly predict functional impairment and lower well-being.

**Methods:**

Forty-five adults aged 18 to 45 years, diagnosed with ADHD according to ICD-10 criteria, were recruited from an outpatient psychiatry clinic. After diagnostic confirmation, participants completed self-report measures routinely used in clinical practice. Executive functioning was evaluated with the *BRIEF-A *, emotional regulation with the *DERS*, functional impairment with the *WFIRS*, and quality of life with the *WHOQOL-BREF.* Correlation and regression analyses explored how executive and emotional regulation difficulties related to daily functioning and overall well-being.

**Results:**

Adults with ADHD in this cohort described significant challenges with both cognitive and emotional aspects of executive functioning. Many reported ongoing difficulties managing time, staying organized, and following through with tasks. Emotional dysregulation was also common, with participants frequently noting that intense emotions disrupted concentration and decision-making. A consistent pattern emerged linking these difficulties to quality of life. Participants who reported greater executive dysfunction also described lower well-being, particularly in the psychological and social domains of the WHOQOL-BREF. Emotional dysregulation showed the strongest association with reduced psychological quality of life, followed by organizational and planning difficulties. Together, these executive domains explained nearly half of the variance in overall QoL scores. Further analysis suggested that the relationship between executive dysfunction and life satisfaction was largely mediated by functional impairment, therefore indicating that difficulties with self-regulation and organization manifest directly as challenges in work, social interactions, and daily living.

**Conclusions:**

Our findings suggest that executive dysfunction, particularly difficulties with emotional regulation and organization, profoundly affects how adults with ADHD experience daily life. Challenges in managing emotions, staying organized, and maintaining focus appear to shape well-being more than core symptoms alone. Supporting patients with strategies that strengthen emotional control and organization can make a meaningful difference in how they feel about their lives as a whole.

**Disclosure of Interest:**

None Declared

